# The Diatom *Staurosirella pinnata* for Photoactive Material Production

**DOI:** 10.1371/journal.pone.0165571

**Published:** 2016-11-09

**Authors:** Roberta De Angelis, Sonia Melino, Paolo Prosposito, Mauro Casalboni, Francesca Romana Lamastra, Francesca Nanni, Laura Bruno, Roberta Congestri

**Affiliations:** 1 University of Rome ‘Tor Vergata’, Department of Industrial Engineering, Rome, Italy; 2 INSTM Consortium, Research Unit Roma Tor Vergata, Rome, Italy; 3 University of Rome ‘Tor Vergata’, Department of Chemical Sciences and Technologies, Rome, Italy; 4 University of Rome ‘Tor Vergata’, Department of Enterprise Engineering, Rome, Italy; 5 University of Rome ‘Tor Vergata’, Department of Biology, Laboratory of Biology of Algae, Rome, Italy; Oregon State University, UNITED STATES

## Abstract

A native isolate of the colonial benthic diatom *Staurosirella pinnata* was cultivated for biosilica production. The silicified cell walls (frustules) were used as a source of homogeneous and structurally predictable porous biosilica for dye trapping and random laser applications. This was coupled with the extraction of lipids from biomass showing potential to fabricate photoactive composite materials sustainably. The strain was selected for its ease of growth in culture and harvesting. Biosilica and lipids were obtained at the end of growth in indoor photobioreactors. Frustules were structurally characterized microscopically and their chemistry analyzed with Fourier Transform Infrared Spectroscopy. Frustule capacity of binding laser dyes was evaluated on a set of frustules/Rhodamine B (Rho B) solutions and with respect to silicon dioxide and diatomite by Fluorescence Spectroscopy demonstrating a high affinity for the organic dye. The effect of dye trapping property in conveying Rho B emission to frustules, with enhancement of scattering events, was analyzed on Rho B doped polyacrylamide gels filled or not with frustules. Amplified spontaneous emission was recorded at increasing pump power indicating the onset of a random laser effect in frustule filled gels at lower power threshold compared to unfilled matrices.

## Introduction

Diatoms are photosynthetic microorganisms that successfully colonize every aquatic habitat; they are environmentally flexible, adapted to the many different aquatic environments in which they live. These highly diverse unicellular or colonial microalgae (1–1000 μm in size) play a significant ecological role, contributing one quarter of global primary productivity and hence to food webs and the carbon cycle [1]. Diatom biomass cultivation has been recently recognized as an excellent biotechnology for high value algal compounds in the nutraceutical context and cosmetics, such as feed, in wastewater treatment as removal agents of pollutants and in the biofuel production [2, 3].

Furthermore, diatoms are principal microorganisms involved in biosilicification, both in terms of the amount of silica they process and for the variety of silicified structures they can create. They produce an external shell, frustule, composed of two halves predominantly made up of silica (valves), which are highly elaborated and display intricate designs at the micro- and nano-meter scale. The species-specific geometry of valves relies on a highly controlled biogenic process allowing symmetries of frustule patterns to be consistently inherited through diatom generations [4–7]. Frustule porous architectures contribute to high surface area for exchange with external environment and display mechanical strength along with efficient luminescence within the visible spectrum [8–10].

In recent years, frustule capacity of manipulating light has been surveyed in a number of diatom species in order to unveil the light-matter interaction and, in particular, that of the complex patterns of pores with incident radiation [11–14]. In the context of diatom biotechnological applications, considerable effort has been devoted to reveal the mechanism of biosilica shell formation and its optical and microfluidic properties, in parallel with the development of procedures to modify frustules into various materials maintaining hierarchical structures [15, 16]. This has provided more opportunities to use biosilica structures in microsystems and other commercial products. Specifically, the quasi-periodic and highly regular features of frustule surface have become very attractive for applications based on optical and photonic properties of biosilica materials. Indeed, interesting possibilities have been opened up for manipulating and exploiting frustules for photonics where a high surface area as well as high porosity are required, e.g. photoanodes in dye sensitized solar cells [17], silica-titania photocatalysts for NOx abatement and air purification under UV radiation and for biomedical applications [18–20], photonic crystals for gas sensing [21] and probes for holographic optical tweezing [22]. In a previous study, we obtained the first experimental evidence of Random Laser (RL) from Rhodamine B doped polymethyl-methacrylate films filled with diatom frustules as scatterers [23]. RL is a special type of laser in which a random assembly of scattering elements dispersed into an optical gain medium replaces the optical cavity of conventional lasers. We observed an incoherent random laser effect using commercial diatomite, fossil frustules, and a mixture of frustules from extant biofilm diatoms to achieve multiple scattering of photons [23]. However, to our knowledge no studies have been published on the effect of selected and homogeneous diatom frustule typologies in RL application, though a better reproducibility of lasing performance could be achieved. In the present work, the aim of exploring homogeneous and structurally predictable biosilica material was pursued by large scale cultivation of a native diatom strain, *Staurosirella pinnata*, isolated from sediments of a Mediterranean coastal lagoon [24–26]. This strain was selected for its rapid growth at high density in laboratory cultures and for the ability to form cell colonies, an intrinsic property that contributed to biomass harvesting by natural flocculation. In addition to biosilica extraction and analysis for dye trapping and RL effect, we evaluated the total lipid content of the diatom biomass obtained. Diatoms are, in fact, good lipid accumulators, rapidly storing lipid droplets on average up to 25% dry weight, with proportions increasing to 45% under nutrient depletion [27–30]. The integration of biofuel production with that of biosilica from residual biomass will allow the employment of frustules with designed and predicted structure for RL application and will significantly contribute to reduce the costs of diatom cultivation [31, 32]. In fact, although diatoms are excellent candidates fulfilling major prerequisites of a sustainable feedstock, there are still several technical challenges hindering their large scale biomass production in an economic and environmentally sound process.

Our results based on the use of biosilica can inspire novel development in photonics, materials science and nanotechnology (environmental monitoring, medical diagnostics and advanced packaging inspection).

## Material and Methods

### Biomass cultivation and analyses

A native strain of the colonial, araphid diatom *Staurosirella pinnata* (Ehrenberg) D.M. Williams & Round was isolated from biofilm material collected from sediments of the Cabras coastal lagoon (Sardinia, Italy, Mediterranean Sea), in September 2010, and maintained in standard Diatom Medium (DM) at 18–20°C, irradiance of 30 μmol photons m^-2^ sec ^-1^ and 12: 12hs L/D cycle.

The stock culture was used as inoculum to cultivate the diatom strain at 20°C and three different growth conditions, combining different light exposure with medium turbulence (air insufflation), to optimize biomass, biosilica and lipid production. Three batch culture replicates for each combination (Table 1) were set up in 400 ml flasks.

**Table 1:** Culture labelling. For each condition three replicates were set up.

Labels	Light:Dark(hours)	Irradiance(μmol photons m-^2^ sec ^-1^)	Turbulence(air bubbling)
**Standard**	12:12	30	-
**HLunmix**	Continuous light	120	-
**HLmix**	Continuous light	120	+

Cell growth was microscopically estimated by cell counting over time. Growth rates were calculated as reported in Guillard [33]. To this end, 3 ml of each culture were sampled every 32–48 hrs. and, after sonication to disrupt cell chains, 3 aliquots, 1ml each, put in counting chambers for microscopically cell enumeration using an inverted light microscope (Zeiss Axiovert 100) equipped with 40 x objective. Cell numbers are reported as average of the three replicate abundances with the relative standard deviations. Liposoluble pigments were extracted from 1ml culture subsamples, every 2 days, by 90% acetone overnight treatment, at -20°C, and the relative absorbance spectra recorded using a Varian Cary 50 Bio UV-VIS spectrophotometer, with readings in the range of 400–700 nm. Absorbance values recorded at 435 nm wavelength (first peak of chlorophyll *a* absorbance), for the HLmix culture, at the exponential growth phase were also plotted against time and cell numbers to correlate pigment absorbance with cell growth and viability [34]. Data analysis was performed using GraphPad Prism version 5.0 for Windows (GraphPad Software, San Diego, CA, USA).

Biomass production at selected growth conditions, HLmix, was performed in 5 liters plastic photobioreactors (PBRs). Three PBRs were inoculated and maintained at continuous illumination in a growth cabinet equipped with white fluorescent lamps (OSRAML 30w/956) and thermostated (20°C). Biomass harvesting was carried out after 7 days using a conical plate centrifuge and total biomass yield (dry weight/time) evaluated after freeze-drying the cultures. A small amount of freeze-dried biomass (1 g) was treated with boiling acids mix (HSO_4_: HNO_3_: H_2_O = 3:1:1) and the resulting cleaned frustules washed in distilled water and freeze-dried. Biosilica culture content was then evaluated in terms of mg product/mg freeze dried biomass. Another aliquot of freeze dried biomass (around 0.3 g) was also extracted with chloroform:methanol solvent mixture (2:1) for 72 hrs.; the lipid phase was separated and then washed with NaCl to remove water; the sample was dried with a Rotavapor Buchi WaterBath B-480 at 45°C and the total lipid quantified.

Epifluorescence microscopy was used to visualize lipid droplets accumulation in living cells during late exponential/stationary phase, at all culture conditions. Samples from each culture were collected and stained with Nile red and observed with a Leitz Aristoplan epifluorescence microscope, 40x objective, using a I3 filter set (excitation wavelength 450–490 nm). Lipid droplet emission was registered at 530 nm.

### Frustule biosilica characterization

Microstructural analysis, using a Field Emission-Scanning Electron Microscope (FEG-SEM, Zeiss Leo Supra 35) was conducted after air drying and gold sputtering a drop of cleaned frustule suspension on stubs. Automatic measurements during SEM sessions were performed on 50 randomly selected frustule valves. Frustules valve axes (apical, transapical) dimensions, striae densities in 10 μm, interstriae distances, numbers of pores per stria and pore morphometry were estimated to evaluate the presence of structural aberrations in the frustules. Fourier Transform Infrared Spectroscopy (FTIR, Perkin Elmer 100) was used for chemical analysis of cleaned frustules. Spectra were acquired in the range 4000–400 cm^-1^, by averaging 32 scans at a resolution of 4 cm^-1^.

### Frustules for dye trapping

The dye-binding analysis was performed using 50 μg/ml Rhodamine B (Rho B) (Sigma-Aldrich) solution and 10 mg/ml frustule suspensions in 10 mM Tris-HCl, pH 8.0 buffer. The following frustules/Rho B w/w ratios were tested in three replicates: 1/1; 5/1; 10/1; 25/1; 50/1; 100/1. After 30 min incubation, at room temperature, the samples were centrifuged (1342 g for 1 min) and the fluorescence spectra of 100 μl supernatant, after addition of 900 μl ddH_2_O, were recorded using a LS50 Perkin Elmer spectrofluorimeter, equipped with a thermostated stirrer cell holder, at 25°C. The excitation and emission bandwidths were both of 5 nm, with the excitation wavelength set at 556 nm. The spectra were recorded from 450 to 680 nm, with a scan speed of 200 nm/sec, maximum emission was recorded at 566 nm.

Rho B binding to frustules, silicon dioxide (SiO_2_) and commercial diatomite powder (DP) was assessed using 10 μg/ml of Rho B solution and suspensions of frustules, SiO_2_ and DP all at the concentration of 1 mg/ml. After incubation (30 min at room temperature), the samples were centrifuged (1342 g/min) and the fluorescence spectra of 100 μl supernatants, after addition of 900 μl ddH_2_O were recorded. The results were plotted using GraphPad Prism version 5.0 for Windows (GraphPad Software, San Diego, CA, USA). The standard deviations were calculated using three or five independent measurements and presented for each type of sample. Epifluorescence microscopy was also performed on 100 μg freeze dried frustules, previously incubated (30 min) with 0.5μg/100μl Rho B solution in 10 mM Tris-HCl pH 8.0 buffer solution, then washed and centrifuged at 1342 g/min. The pellet was observed using a Leitz Aristoplan epifluorescence microscope equipped with M2 filter set (excitation wavelength at 546 nm) and 40x objective.

### Frustules for Random Laser

#### Sample preparation

A frustule suspension was used as filler. The gels were prepared with 10% and 20% of polyacrylamide using 30% acrylamide/N,N'-methylene-bis acrylamide solution (29:1) (Sigma-Aldrich, Milan, Italy), 3μl of 10% ammonium persulfate (APS) (BioRad) solution, with or without 1% w/v of frustules, 2.5μl of 0.1mg/ml Rho B solution (Sigma–Aldrich, Milan, Italy) and ddH_2_O for a final volume of 65μl. The polymerization was performed using 1 μl of tetramethylethylenediamine (TEMED) (BioRad) in O-ring of 5 mm diameter and 1mm of thickness in order to obtain gels with the same dimensions.

#### Confocal Laser Scanning Microscope analysis

Emission spectra of frustule water suspensions and those of 10% and 20% acrylamide gel filled with frustule suspension (1% w/w), were detected by means of spectral analysis (SA) using an Olympus FV1000 Confocal Laser Scanning Microscope (CLSM) through laser excitation: diode lasers (405 and 635 nm), argon laser (488 nm) and helium neon laser (543 nm). Emitted light intensities were measured at intervals of 5 nm across the total spectrum and respective intensity profiles were registered. Spectra were acquired using 2D reconstructions of valves after optical sectioning of the Region Of Interest (ROI, step size 1 mm), background signal, due to the glass slide, was calculated and subtracted from the final spectra.

#### Random Laser measurements

Photoluminescence (PL) of the Rho B was measured at room temperature by using the frequency doubled output (532 nm) of a 10 Hz, Q-switched Nd:YAG laser as excitation source. The pump pulse duration was 6 ns. A half-wave plate and a Glan–Taylor polarizer prism were used to control the laser power, which has been measured with an energy meter. The laser spot area on the sample was 16 mm^2^. The emission was collected in front configuration with a fiber bundle trough a lens and analyzed by a spectrometer (OceanOptics HR4000).

## Results and Discussion

### *Staurosirella pinnata* cultivation and biomass yields

**Fig 1:**
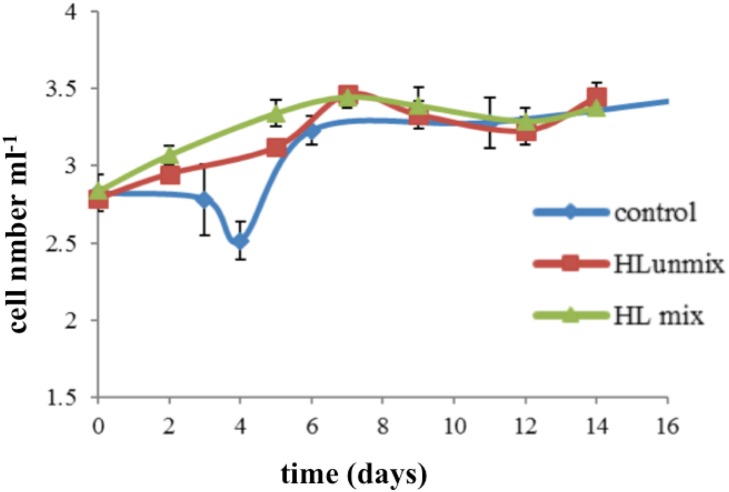
Growth curves. For the three culture conditions tested growth curves were registered over the 16 day experiments. Cell concentration is calculated as the mean value of the three culture replicates set for each growth conditions and expressed as log_10_ cells mL^-1^. Standard deviations of the mean values are reported.

Growth curves registered at the three culture conditions tested evidenced that continuous and high light (HLmix and HLunmix cultures) favored cell division and initial biomass development in culture with growth rate estimates of 0.13, 0.21 and 0.22 for standard, HLunmix and HLmix cultures, respectively (Fig 1). HLmix cultures showed no lag phase with exponential growth occurring soon after inoculation, and maximum cell concentration (2.8x10^3^ cells mL^-1^) was achieved at day 7.

**Fig 2:**
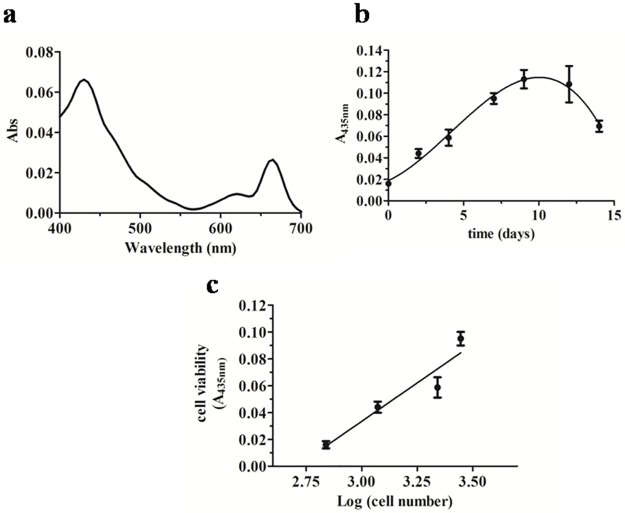
Correlation analysis between liposoluble pigment absorbance and *S*. *pinnata* growth at HLmix conditions. a) UV-vis absorbance spectrum of culture liposoluble extract obtained at the exponential phase; b) Non-linear polynomial fitting of absorbance values recorded at 435 nm over growth time; c) Linear regression between 435 nm absorbance values and Log cell numbers at days 0, 2, 4 and 7. Bars represent SD between culture replicates.

This sharp growth also reflected the effect of air insufflation that allowed colony mixing and an efficient use of light for the photosynthetic process. The benthic lifestyle of this diatom and its tendency to settle at the bottom of the flask probably affected the initial cell growth at the static condition. Although maximal cell concentrations were similar under the three diverse growth methods tested, HLmix cultures showed continuously increasing densities up to the maximal at day 7, indicating a more stable growth trend that led us to the selection of this conditions for the following growth experiment and frustule/biosilica production at larger scale using indoor closed photobioreactors. In addition, spectrum analysis of the liposoluble extracts of HLmix cultures, at different phases of the cell growth, showed two absorbance maxima at 435 and 665 nm (Fig 2a). In particular, we observed a correlation between the absorbance values at 435 nm, attributed to chlorophyll *a*, and the growth phases as shown in Fig 2b. A sigmoid trend of the absorbance values at 435 nm over time was also observable, suggesting the possibility to monitor the photosynthetic/metabolic state and viability of cells, indirectly, by means of this spectral analysis. Therefore, a correlation between the cell numbers and the 435 nm absorbance at day 0, 2, 4, 7, 9, 12 and 14 of growth was performed, recording a good linear regression (R^2^ = 0.849) up to day 7 of HLmix growth (Fig 2c). These findings suggest that spectrophotometric analysis data were in agreement with cell counting obtained by microscopy analysis and could represent a reliable and quick method for monitoring the cell viability and metabolic activity in culture in order to identify the state of the cell culture and growth and consequently suggesting proper biomass harvesting time.

**Fig 3:**
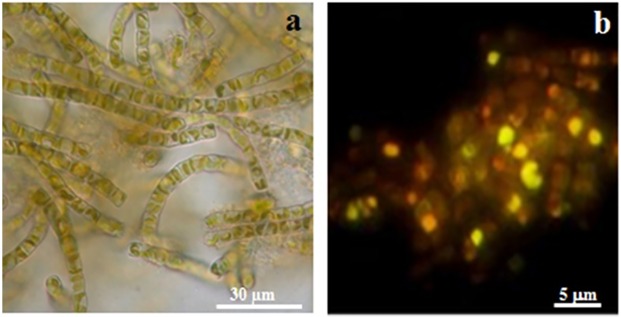
Micrographs of *Staurosirella pinnata*. (a**)** Bundles of filamentous colonies of HLmix cultures observed in light microscopy, bar = 30 *μ*m *(*b) Yellow fluorescence of oil droplets in HLmix cells stained with Nile red. Bar = 5 *μ*m.

Finally, cells in the HLmix cultures appeared in a healthy state at the light microscope, showing no shrinkage of plastids and active cell reproduction, with daughter cells visible within the frustules of parental cells (Fig 3).

Thus, the diatom was grown in the three photobioreactors for 7 days at HLmix conditions. At day 8, the cultures were stopped (late exponential/beginning of the stationary phase), the biomass was collected by settling and centrifuging, then pooled together to be subsequently characterized. Biomass weight (FW) was calculated after dewatering (fresh weight, FW = 6.25 ± 0.11 g/L) and freeze-drying (dry weight, DW = 200 ± 0.1 mg/L); the daily productivity reached 28.75 ± 0.13 mg/L/day. These biomass yields and values of productivity are comparable with those obtained for diatoms and other microalgae mostly used in biotechnological applications [30]. Biomass lipid accumulation was monitored with CLSM in flask culture samples stained with Nile red, thanks to the dye affinity for cellular neutral lipids. Maximal content of lipid droplets was observed at the beginning of stationary phase in the HLmix culture as shown in Fig 3b. Total lipids accounted for 14% of freeze dried biomass, with a lipid productivity of 4.5 mg/L/day ± 0.35. The value of total lipid content we found is in the range reported for the same diatom by Renaud et al. [35] that referred to the species as *Fragilaria pinnata*, and for other species as *Nitzschia* sp. [35] and *Amphiprora paludosa* [36] grown in similar (non optimized) culture conditions. It is known that an increase in biomass lipid content can be obtained under cell nutrient starvation, e.g nitrogen, silicate sources [28, 30], but in this experiment, we did not apply nutrient depletion to preserve the biosilica porous integrity and homogeneity of all the frustules produced in culture since nutrient unbalances could lead to anomalies in silica deposition and ultimately alter frustule ultrastructure. Recent advances in microalgal intensive cultivation highlighted the potential to increase the cell lipid content by physical methods, growth under monochromatic Light Emitting Diodes (LEDs) [37] or by genetic modification (lipid biosynthesis mutants) [38]. These methods could avoid the impact of nutrient starvation on frustule structure, that affect frustule application in the production of photoactive material. To this end, the chance to profitably extract biosilica and lipids from the same biomass become particularly important for a follow-up in the production of diatom biomass for optical applications in nano-device fabrication.

After hot acid cleaning, to remove most of the organic material from biosilica, we calculated the weight of total biomass frustules that attained the value of 0.27± 0.07 g, representing 16.7% of the biomass DW.

### Structural and chemical characterization of frustules

**Fig 4:**
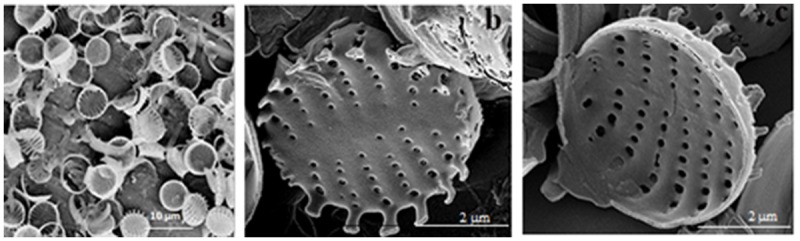
SEM micrographs of biosilica material. Single valves obtained with hot acid cleaning (a); rows of circular pores are visible on valve close ups in outer (b) and inner view (c).

Acid cleaned material analysis with SEM showed that frustule valves mostly appeared singly, indicating that the cleaning treatment allowed the junction between adjacent cells of the chains to break, eventually providing a homogeneous assembly of roundish/elliptical valves (4–5 *μ*m in diameter) bearing spatulate linking spines at their margins (Fig 4). External valve surfaces had pore rows (striae) of 5–6 circular areolae, whose diameter slightly increased marginally. The cleaning treatment also removed pore cribra (sieve partially occluding the pore internally) from the internal valve surface.

No significant aberration of pore patterns was evidenced after analyzing by automatic measurements of the most important ultrastructural parameters of valves and pores on a set of 50 valve images at SEM. 3–4% frustules only presented slight aberrations (incomplete pore margins) in pore patterns and an increase in whole valve size, probably due to the culture conditions applied. This indicates that large scale *S*. *pinnata* cultures are a good source of homogeneous and structurally predictable porous biosilica when a specific and uniform porosity is required.

**Fig 5:**
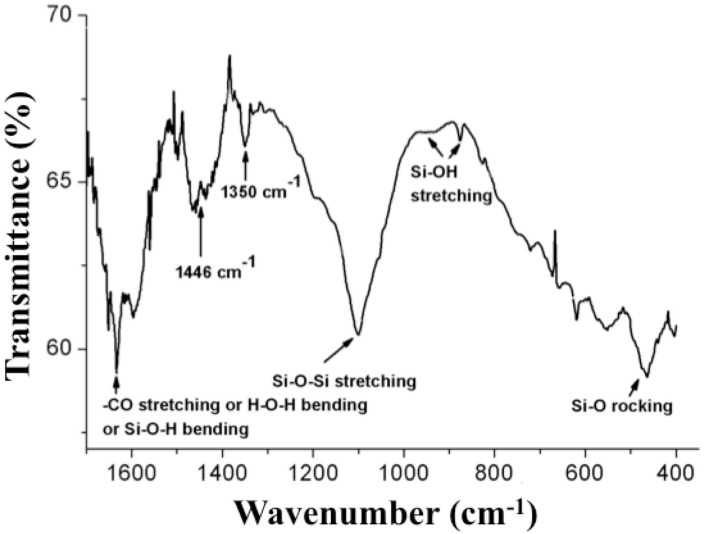
FTIR of acid cleaned *S*. *pinnata* frustules.

The FTIR spectrum of acid cleaned *S*. *pinnata* frustules is reported in Fig 5. According to the literature [39–41], the sample showed absorption bands characteristic of silica. In details, the peak at 1100 cm^-1^ corresponds to Si-O-Si anti-symmetric stretching mode, the absorption around 466 cm^-1^ is associated to Si-O rocking vibration. The peaks at 930 and 875 cm^-1^ are attributable to Si-OH stretching. Moreover, the peak at 1632 cm^-1^ could be attributed to several vibrations: -CO stretching for primary amides of residual proteins (i.e. silaffins) embedded within diatom biosilica, or H-O-H bending of adsorbed water or Si-O-H bending [42, 43].

The bands located at 1446 and 1350 cm^-1^ are associated to side chain absorption in proteins probably closely attached or even embedded into diatom cell walls. In detail, these bands correspond to bending vibrations of CH_2_ and CH groups, respectively [44]. Thus, the FTIR analysis carried out on acid cleaned frustules of *S*. *pinnata*, grown at HLmix conditions, detected several functional groups that can act as binding sites for organic molecules.

### Frustules as potential dye traps

Porous silica has been extensively used in industry due to its low cost and the different pore structure with multi-scale controlled pore size [45]. In addition, photoactive nanostructured material production represents an interesting area that opens the way for useful composite materials with desirable optical properties.

**Fig 6:**
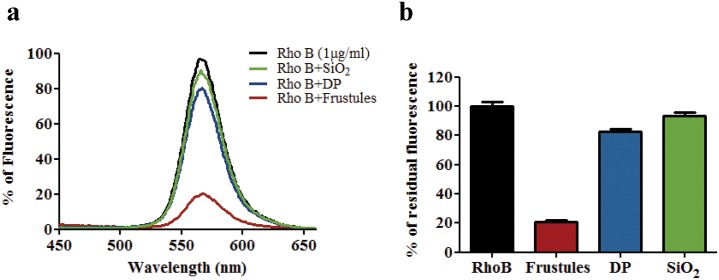
Fluorescence analyses of the Rho B binding to the frustules. a) Fluorescence of 5 μg/ml of Rho B solution after incubation at different frustules/Rho B w/w ratios: 0, 10, 25, 50 and 100, in 10 mM Tris-HCl, 8.0 pH, buffer. λ_ex_ at 556 nm and λ_em_ from 450 to 680 nm; b) 50 μg/ml of Rho B solution after 30 min of incubation with 0.5 mg of frustules and centrifugation; c) Plot analysis of the % of fluorescence variation (% (Fo-F)/F_0_) of the Rho B solution at 566 nm after incubation at different frustules/Rho B w/w ratios; bars represent SD values; d) Fluorescence micrograph of the frustules after incubation with 10 μg/ml Rho B and washing; scale bar 10 μm.

To investigate the possibility to produce a functional biocomposite material containing laser dyes as one of its constituents, several experiments were performed using fluorescence spectroscopy. In Fig 6a the fluorescence spectra of 5 μg/ml RhoB solution using different frustules/Rho B w/w ratios are shown. The presence of frustules in the Rho B solution decreased the fluorescence intensity in a concentration dependent manner (Fig 6a and 6b), due to the adsorption of the fluorophore to the frustules. Thus, frustules showed very good affinity for organic dye molecules like Rho B.

The analysis of the dye binding at different frustule concentrations showed that the dye-frustule binding occurred with a sigmoid curve (Fig 6c), with an apparent K_d_ of 0.1461±0.010 mg/ml (ligand concentration that binds to half the receptor sites at equilibrium), a maximum number of binding sites of 71.05±3.95 and a Hill slope of 2.729±0.519. These results indicated the presence of multiple binding sites with positive cooperativity. Thus, the binding of organic dye to the frustules may be due to both physical and chemical factors, such as adsorption of the dye into the nanoporous structure of frustules and chemical interactions between the dye molecules and the functional binding sites present on the silica surface, in agreement with the FTIR data analysis. Epifluorescence microscopy analysis of frustules showed that 30 min of incubation with Rho B and washing led to fluorescent structures without visible morphological alterations of frustules (Fig 6d).

**Fig 7:**
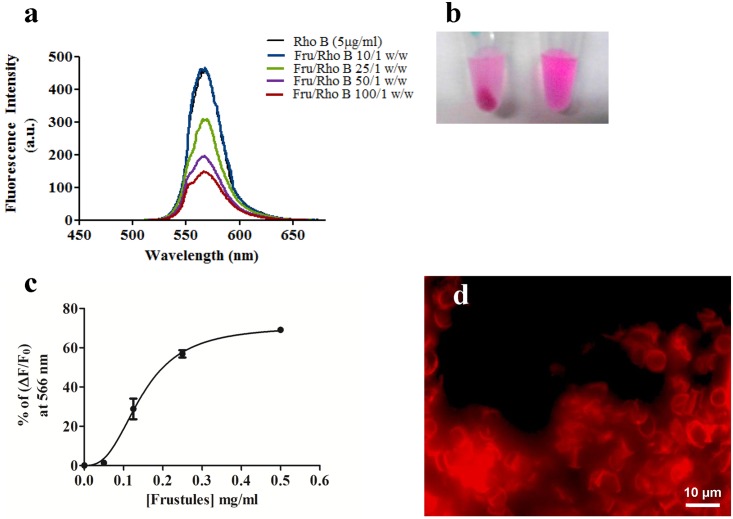
Different behavior of silica structures in the Rho B binding. **a)** Fluorescence spectra of 1 μg/ml of Rho B solution after 30 min of incubation at room temperature with 0.1 mg/ml of frustules, DP or SiO_2_ and centrifugation; **b)** % of residual fluorescence at 566 nm of 1 μg/ml Rho B solutions after incubation as described. The values were normalized in respect to the fluorescence of Rho B solution alone. Each bar represents SD of three independent experiments.

Moreover, in order to investigate on the relevance of the homogeneous nanoporous structure of frustules as dye traps, a comparison of the dye-binding ability between frustules and silicon dioxide (SiO_2_) and commercial diatomite powder (DP) was performed by fluorescence measurements. Fig 7a shows the residual fluorescence signals of the Rho B solutions after 30 min incubation with frustules, DP and SiO_2_.

Incubation with the frustules caused a decrease of 79.29 ± 1.49% fluorescence of the dye solution. Conversely, fluorescence decrements of only 17.38 ± 1.89% and 6.418 ± 2.46% were detected for the dye solution incubated with DP and SiO_2_, respectively (Fig 7b). These results indicate that the dye trapping is related to the nanostructure of frustules and to the homogeneity of the biosilica obtained from the diatom culture. It has to be noted that commercial DP has been subjected to a calcination process that could have a relevant influence on the decrease of the binding properties of organic molecules on the silica surface, such as on the absence of intrinsic photoluminescence of the DP [23].

The above dye-binding property of the frustules could be used for the fabrication of devices for the dye-trapping or for slow-releasing of fluorescent chromophores and, eventually, for their conjugates with bioactive molecules for diagnostic and therapeutic applications.

### Frustule photoluminescence silencing

**Fig 8:**
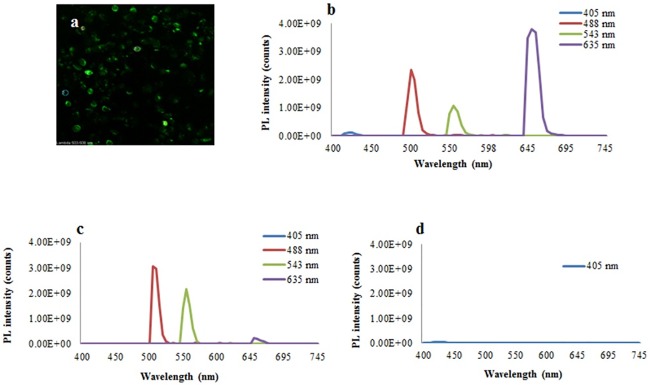
Photoluminescence analysis of frustules by SA-CLSM. a) 2D reconstruction of frustule signal after argon laser (488) excitation, selected ROIs are also shown. b) Emission spectra recorded through laser excitation at 405, 488, 543, 635 nm for aqueous frustule suspension and for composites frustule of 10 (c) and 20% (d) AA matrices.

In our previous study frustule intrinsic photoluminescence (PL) was supposed to alter the random laser effect [23]. In order to reduce the PL effect, probably due to silanolic groups present on the surface of acid cleaned frustules, the frustules were dispersed in polyacrylamide (AA) gels and their PL assessed by means of Spectral Analysis at CLSM (SA-CLSM). Fig 8 shows the emission spectra of the frustule solution in H_2_O_dd_ (b) and after embedding of the frustules in gels matrices of 10% (c) and 20% (d) AA. The total quenching of frustule photoluminescence was obtained using a matrix of 20% AA.

### Random Laser effect of composite frustules-AA matrix

The observed dye trapping property of the frustules could have the effect of conveying the Rho B emission to the intricate pore pattern, thus enhancing the scattering events, which provide the required feedback mechanism for random laser (RL) [46, 47].

**Fig 9:**
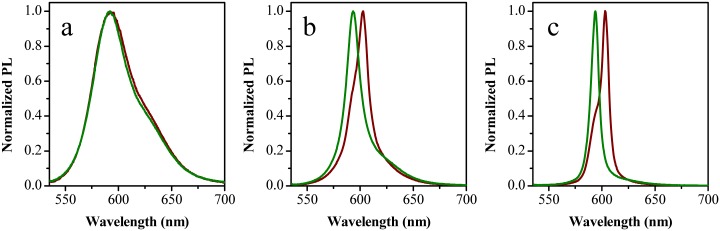
Normalized PL spectra of the unfilled (brown lines) and filled (green lines) samples at a) low, b) intermediate and c) high pump power.

In a RL experiment, the PL band shape of the sample is investigated as a function of the excitation power. Above a certain threshold, the spectrum narrows with the increasing of pump power, indicating the onset of a process, which is known as amplified spontaneous emission (ASE). We studied the RL effect of two types of matrices doped with the same amount of Rho B. The first was a 20% acrylamide gel (referred to as unfilled) and the second was a 20% acrylamide gel filled with diatom frustules (referred to as filled). The normalized PL spectra are reported in Fig 9.

**Fig 10:**
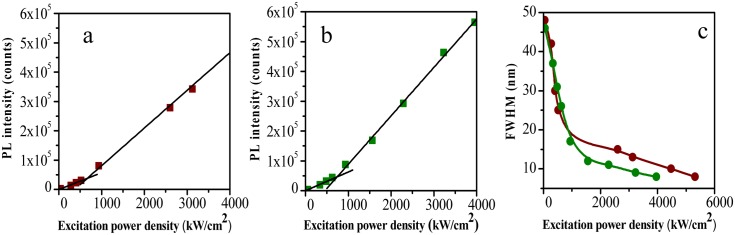
Power dependency of the PL maximum intensity. PL peak intensity as a function of the excitation power for the a) unfilled and b) filled sample. Black lines are linear fits of the data. c) Power dependency of the FWHM for the unfilled (brown circles) and filled (green circles) samples.

At low excitation energy, well below the threshold, the emission spectrum is due only to the spontaneous emission of the dye for both types of samples. The result is a broad-band spectrum with peak position at 592 nm and full width at half maximum (FWHM) of 47 nm as shown in Fig 9a. The PL spectra of the two gels just above the threshold are presented in Fig 9b. In both cases, the onset of a RL phenomenon is clearly visible as a narrowing of the PL spectrum. Interestingly, the narrow bands are peaked at different wavelengths namely 594 nm for the filled and 603 nm for the unfilled sample, respectively. The FWHM is reduced to 17/18 nm for the filled/unfilled gel. Finally, Fig 9c shows the spectra at high pump power (4x10^3^ kW/cm^2^) for the two samples. The peak wavelengths are the same as seen in Fig 9b, the spectral widths are further reduced and the FWHM is only 8/10 nm for the filled/unfilled gels. The difference in the peak wavelength between the two samples does not correspond to a difference in the absorption edge of the two materials, so it cannot be ascribed to a self-absorption process. The different peak wavelength is due to the influence of diatom frustules on the gel matrix. The power dependency of the PL maximum intensity as a function of the pump power (input-output characteristic) is reported in Fig 10a and 10b for the unfilled and filled sample, respectively.

The laser threshold was estimated with linear fits of the input-output characteristic with good confidence (R^2^ = 0.99). In the case of the unfilled sample it is equal to 531 kW/cm^2^, while for the filled one the value is reduced to 455 kW/cm^2^. This result is not surprising, since the laser threshold is strongly correlated with the scattering properties of the matrix. In a previous paper, we showed that the addition of diatom frustules is a viable option to control the scattering properties and random lasing in a dye doped PMMA matrix [23]. PMMA is a transparent polymer that does not show scattering properties by itself. In this material, the random lasing phenomenon is controlled by the concentration of scattering particles, this being impossible without them and always occurring at the same wavelength. By contrast, in acrylamide gel RL is achieved even without the addition of scattering particles, thanks to the peculiar properties of the gel structure. However, the diatom frustules are able to modify the characteristics of the RL phenomenon, by changing the lasing wavelength and lowering the power threshold. Fig 10c reports the FWHM as a function of the pump power for both gels. The threshold estimated by FWHM, not shown in figure, has the same value obtained with the previous method. The difference between the two samples is also evident in this graph, where the spectral narrowing of the filled sample requires less pump energy: it reaches 8 nm at 4MW/cm^2^, while the FWHM of the unfilled sample is still 10 nm. In addition, the unfilled gel narrows down to 8 nm but only at about 6MW/cm^2^. It is important to stress that we could not reduce this value further due to sample degradation at such a high power density. These results show that in a Rho B doped acrylamide gel it is possible to achieve RL effect at a different wavelength and with a lower power threshold by adding diatom frustules to the gel.

**Fig 11:**
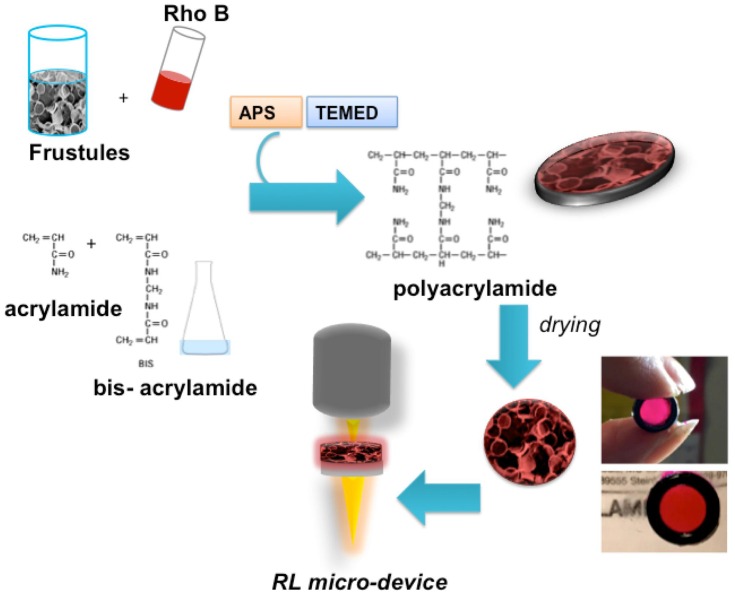
Schematic representation of the steps in the synthesis of the composite material for RL photonic devices. A picture of the composite frustule-AA matrix is shown.

The results here presented show that *S*. *pinnata* frustules are natural nano-porous silica structures characterized by ideal micro-environment that can generate special optical effects of fluorescent probes, as well as RL effect. The composite material, obtained by embedding of these homogeneous natural silica structures into acrylamide polymer, shows the optical property to produce RL effect. Acrylamide was chosen as it is a hydro-soluble molecule, which polymerizes quickly and easily, without using particular equipment. In addition, acrylamide is able to reduce the intrinsic photoluminescence of the diatom frustules, as we have demonstrated in this study. Although, the acrylamide is a toxic molecule, its polymeric form is not and the proposed composite is obtainable by simple mixing of water-solutions and frustules, as shown in Fig 11. Therefore, the non-toxicity of the final product, its ease of handling, resistance over time and ability to promote RL effect make it a possible candidate material for the fabrication of kit for micro-laser devices at low cost.

## Conclusions

We demonstrated that a native isolate of the diatom *Staurosirella pinnata* can be grown in laboratory culture with the aim to produce homogeneous and structurally predictable porous biosilica for photoactive material fabrication. Cultures reached maximal densities rapidly and and were easily harvested by cell settlement due to cell filament formation. A correlation between cell accumulation over time and photosynthetic pigment absorbance was also found allowing to quickly monitor the cell viability and metabolic activity and ultimately to identify proper biomass harvesting time. Frustule biosilica maintained its structural integrity over growth time providing amenable material in terms of quantity, structural homogeneity and thus optical properties. Lipids were also extracted from the same diatom biomass to evaluate the potential to integrate biosilica production with that of diatom lipids for a further diatom biomass exploitation in the field of biofuel and food/feed ingredient production.

The random laser effect induced by *S*. *pinnata* frustules in the acrylamide matrices together with their high capacity in trapping Rhodamin B, is important for different applications. On one hand, the specific entrapment property observed in the studied frustules can be exploited to preserve dye molecules directly attached to biosilica, offering the possibility of planning and modulating the dye release by merely adding a specific solvent. Alternatively, frustule capability of absorbing a specific dye can be exploited for removing dyes and other pollutants from water with possible application in water treatment and purification. Moreover, the dye entrapped by selected and homogeneous frustules can produce a more efficient random laser effect opening the way to the use of selected diatom frustules for manufacturing composite materials useful in the field of photoactive nano-structured materials for integrated micro-laser and luminescent coatings and paints for surfaces of arbitrary shapes.
